# Supplementary material for: Large-magnitude events unlikely in induced earthquake sequences
Source: Nat Commun. 2026 Apr 29;17:4192. doi: 10.1038/s41467-026-72219-9 (PMC13153268; doi:10.1038/s41467-026-72219-9)
Supplement: Supplementary file 1 — Supplementary Information [file 41467_2026_72219_MOESM1_ESM.pdf]

Supplementary Information

Large-magnitude events unlikely in induced earthquake sequences

Linxuan Li[1], Kyungjae Im[1], Jean-Philippe Avouac[1]
[1]Division of Geological and Planetary Sciences, California Institute of Technology, Pasadena, CA, 91125, USA
*Corresponding author: Linxuan Li (lxli@caltech.edu)

Contents of this file
Supplementary Text 1
Supplementary Figs. 1 to 15

Supplementary Text 1 | Real-time monitoring approach

We propose a framework for real-time seismic hazard monitoring to inform injection operations dynamically as seismicity evolves. A defining feature of this approach is that, at any given time, only prior events are available, unlike the retrospective analyses, where p_{LRT} incorporated both past and future data (for example, Supplementary Fig. 6). We demonstrate the framework using the Decatur CO_2 storage site in the United States and the Basel enhanced geothermal field in Switzerland. For Basel, we use a high-resolution catalog[1] that lowers the magnitude of completeness by approximately 0.2 compared to the catalog employed in earlier analyses. We did not use this catalog previously because it lacks event location information.

The first step is to assess whether the sequence follows a GR or TGR distribution. To do this, we calculate the temporal evolution of p_{LRT} using both expanding and sliding windows. In the expanding window, the analysis begins with the first recorded event and progressively includes subsequent events. In the sliding window, only the temporally nearest events are considered.

For genuine TGR cases, a decreasing trend in p_{LRT} is expected as the tail becomes more fully sampled. Comparing p_{LRT} trajectories of expanding- and sliding-window analyses helps distinguish genuine GR conformity from statistical artifacts related to limited sample sizes. For example, in Basel, the sliding-window p_{LRT} prior to the mainshock remains high. If this were solely due to insufficient sampling, the earliest expanding windows over the same time intervals would also yield high p_{LRT}. Instead, these expanding windows show consistently low p_{LRT}, indicating a real temporal shift in the MFD.

The second step is to forecast the maximum magnitude over a future interval. This forecast is based on the MFD model (type and parameters) estimated from events recorded so far, combined with a prediction of the earthquake occurrence number. Integrating these components enables updating the maximum-magnitude forecast in real time as new events are recorded. This procedure differs from the temporal evolution of m_{max} shown in Supplementary Fig. 6, where estimates incorporate events within the forecast window. In practice, different operational strategies can be incorporated into the rate model[2,3] to inform risk management and optimize injection plans.

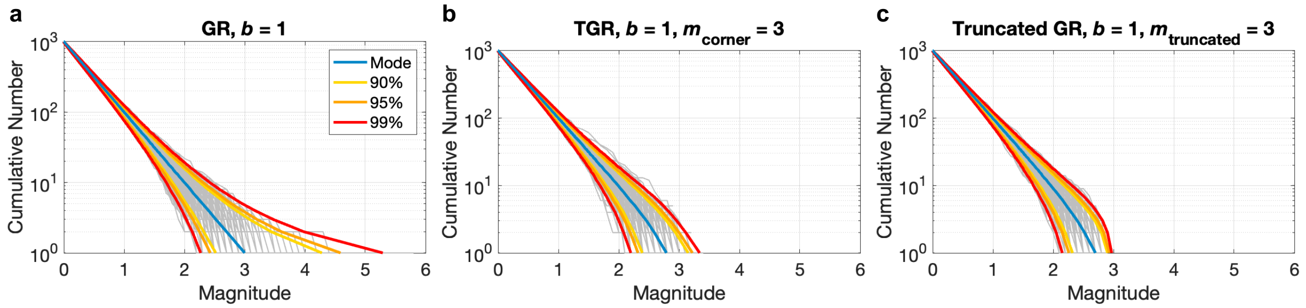

Supplementary Fig. 1 | Confidence intervals of magnitude–frequency distributions. **a,** Unbounded Gutenberg–Richter (GR) distribution with a catalog size of 1000, and a b-value of 1. **b,** Tapered GR (TGR) distribution with a catalog size of 1000, a b-value of 1, and an m_{corner} of 3. **c,** Truncated GR distribution with a catalog size of 1000, a b-value of 1, and an $m_{truncated}$ of 3. Gray curves show 1000 random catalogs for reference. The mathematical form of the truncated GR distribution is[4,5]: $P(\geq m) = \begin{cases} \frac{10^{-bm}-10^{-bm_{truncated}}}{1-10^{-bm_{truncated}}}, m \leq m_{truncated} \\ 0, m > m_{truncated} \end{cases}$.

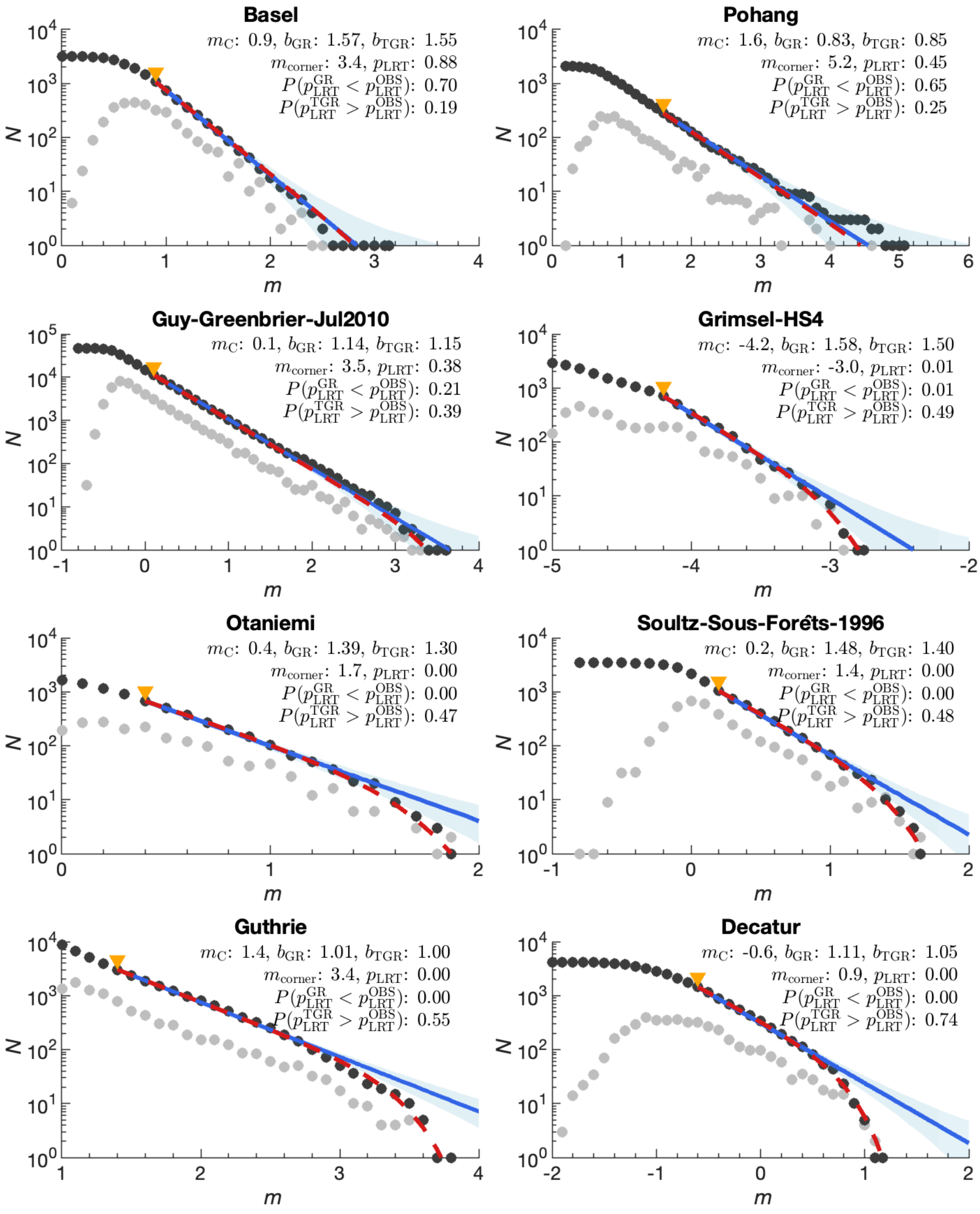

Supplementary Fig. 2 | Examples of the analysis of magnitude–frequency distributions. Gray circles indicate the number of events within each magnitude bin (binned counts), while black circles show the cumulative number of events with magnitudes greater than or equal to each value. The blue solid line represents the best-fitting Gutenberg–Richter (GR) distribution, with the shaded area indicating the 90% confidence interval. The red curve denotes the best-fitting tapered Gutenberg–Richter (TGR) distribution. The yellow triangle marks the magnitude of completeness.

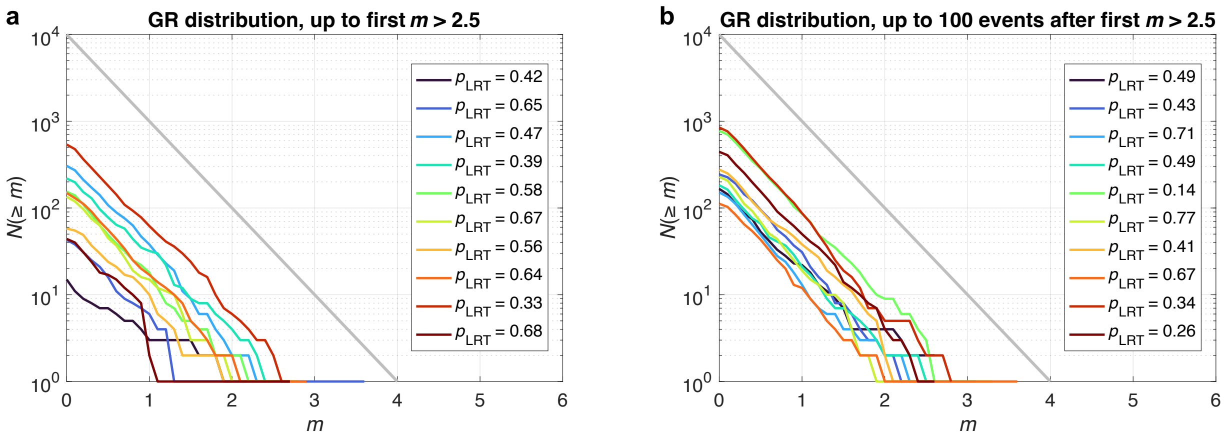

Supplementary Fig. 3 | Sequence termination does not lead to misclassification of an inherently Gutenberg–Richter (GR) distribution as tapered Gutenberg–Richter (TGR). We simulate earthquake sequences following a GR distribution with a *b*-value of 1, a catalog size of 10,000, and a minimum magnitude (equal to the magnitude of completeness) of 0. The theoretical distribution is shown by the gray line. Two scenarios are considered: **a,** the catalog is truncated upon the occurrence of the first event with magnitude >2.5, and **b,** the catalog is extended to include the subsequent 100 events after that occurrence. The second scenario mimics cases where fluid injection is halted after a relatively large event (set to 2.5 here), but seismicity continues for some time thereafter. For each scenario, 10 random realizations are generated (shown as colored curves), and likelihood-ratio tests are applied to classify each sequence as GR or TGR (indicated in the legend).

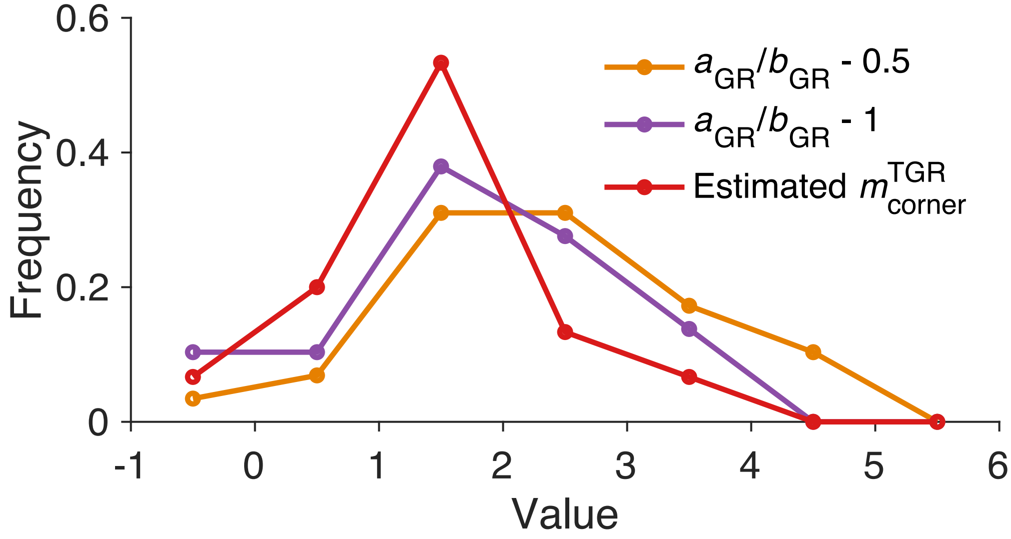

Supplementary Fig. 4 | The absence of tapered Gutenberg–Richter (TGR) cases with large corner magnitudes (m_{corner}) is not due to methodological limitations. The likelihood-ratio test can identify TGR catalogs only when the distribution tail is sufficiently sampled. The lack of TGR cases with $m_{corner} >$ 3 could, in principle, arise if such catalogs are misclassified as Gutenberg–Richter due to insufficient sampling, as in most cases the observed maximum magnitudes are below 4. To evaluate this possibility, we compare the distribution of estimated m_{corner} for TGR cases ($p_{LRT} < 0.05$; red) with the distribution of $a_{GR}/b_{GR} - 0.5$ (orange) and $a_{GR}/b_{GR} - 1$ (purple) for 29 cases (after excluding small-scale experiments). The likelihood-ratio test identifies TGR catalogs with ≥90% probability when $a_{GR}/b_{GR} - m_{corner} > 1$, and with ≥50% probability when $a_{GR}/b_{GR} - m_{corner} > 0.5$. The results indicate that if TGR distributions with larger m_{corner} values were as common as those with m_{corner} between 0 and 3, they would have been detected. Therefore, the observed m_{corner} distribution likely reflects the typical range for induced seismicity sequences following a TGR model.

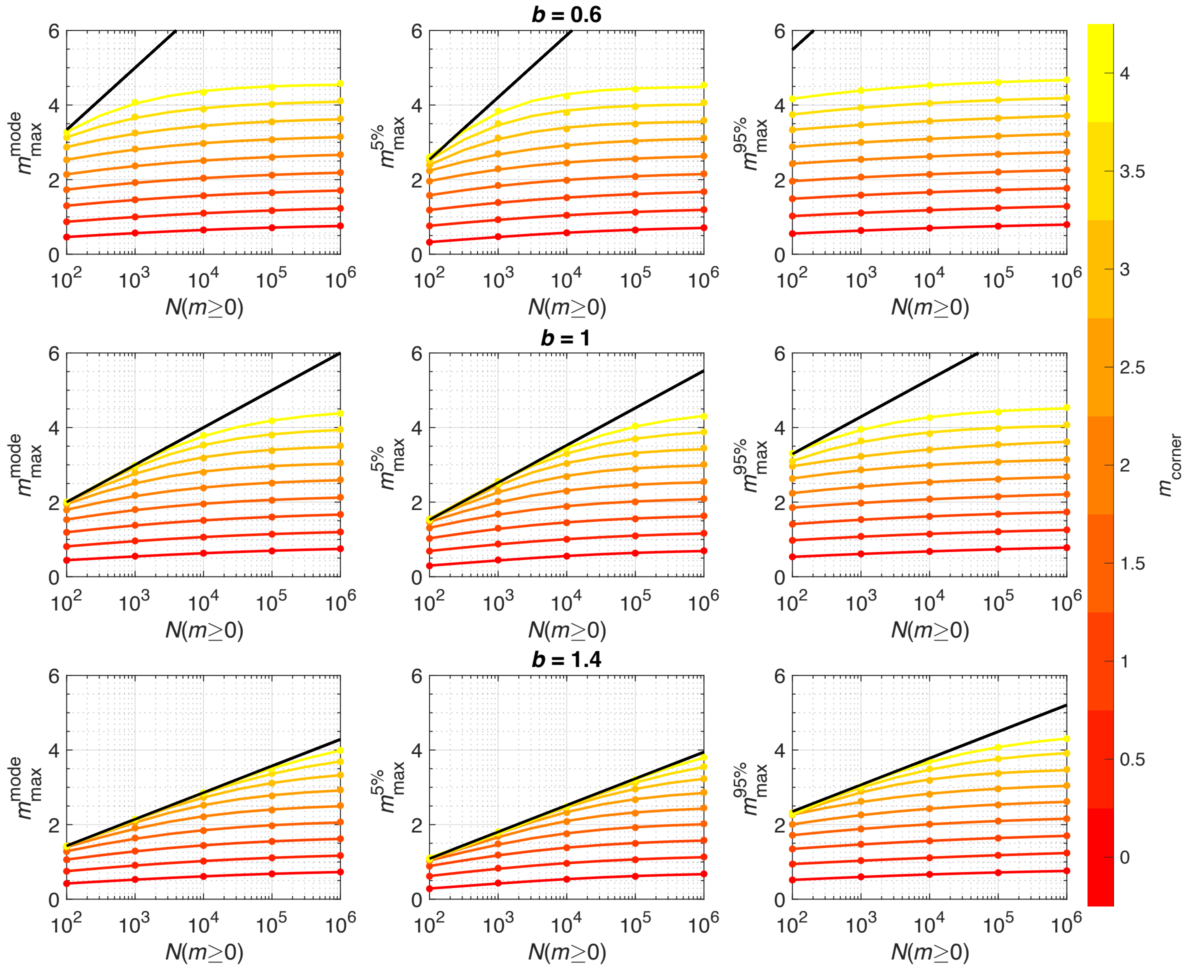

Supplementary Fig. 5 | The maximum magnitude increases rapidly with catalog size for Gutenberg–Richter (GR) catalogs, but shows only a subtle increase for tapered Gutenberg–Richter (TGR) catalogs once the corner magnitude is reached. The mode and the 5th and 95th percentiles of the maximum magnitude predicted by the GR distribution is shown by black lines (Methods, Eqs. 3 and 4). The values predicted by TGR distributions under different catalog sizes (x-axis), corner magnitudes (color), and b-values (panels) are shown in yellow to red circles and curves. Circles represent empirical results obtained by generating a large number of random synthetic catalogs. Curves show logistic fits to the empirical results according to the equation $m_{\max} = \frac{K_{lgt}}{1+e^{-\alpha_{lgt}\left(\log_{10} N(m\geq 0)-\gamma_{lgt}\right)}}$, where K_{lgt}, α_{lgt}, and γ_{lgt} are estimated via nonlinear regression on the synthetic data.

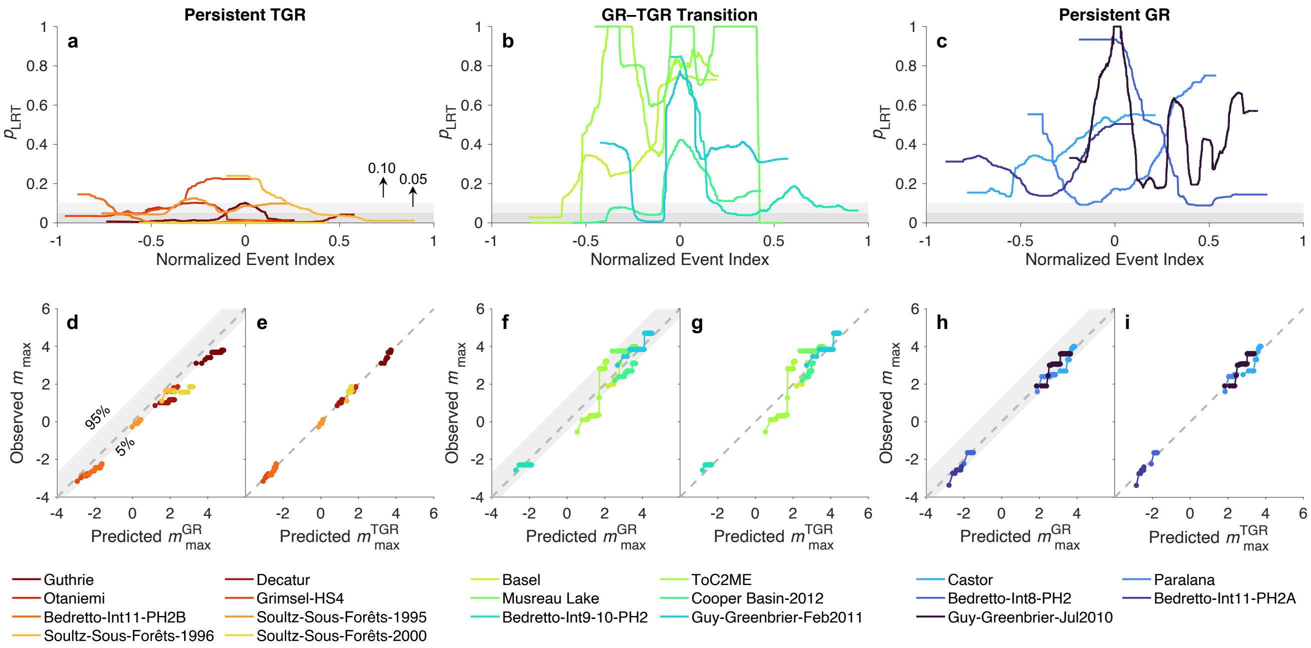

Supplementary Fig. 6 | Adherence to the Gutenberg–Richter or tapered Gutenberg–Richter distribution can vary over time. a, b, c, Temporal evolution of the likelihood-ratio test p-value (p_{LRT}). **d, f, h,** Temporal evolution of the observed maximum magnitude versus the maximum magnitude predicted by the GR distribution. The gray area represents the 90% confidence interval for the maximum magnitude under a GR distribution with b-value of 1 (Methods, Eq. 4). **e, g, I,** Temporal evolution of the observed maximum magnitude versus the maximum magnitude predicted by the TGR distribution. For simplicity, we do not show the confidence interval for the predicted maximum magnitude because it depends on catalog size, b-value, and corner magnitude and therefore strongly varies across sequences and time windows. Panels a, d, and e show cases with quasi-persistent TGR behavior; b, f, and g represent cases that experience transitions between GR and TGR; and c, h, and i display cases that follow persistent GR behavior over time. Maximum-magnitude predictions are derived from expanding windows of 100 events, using parameters from the full catalog. The plotted value represents the modal estimate; results using local-window parameters or the 5th and 95th percentiles are consistent.

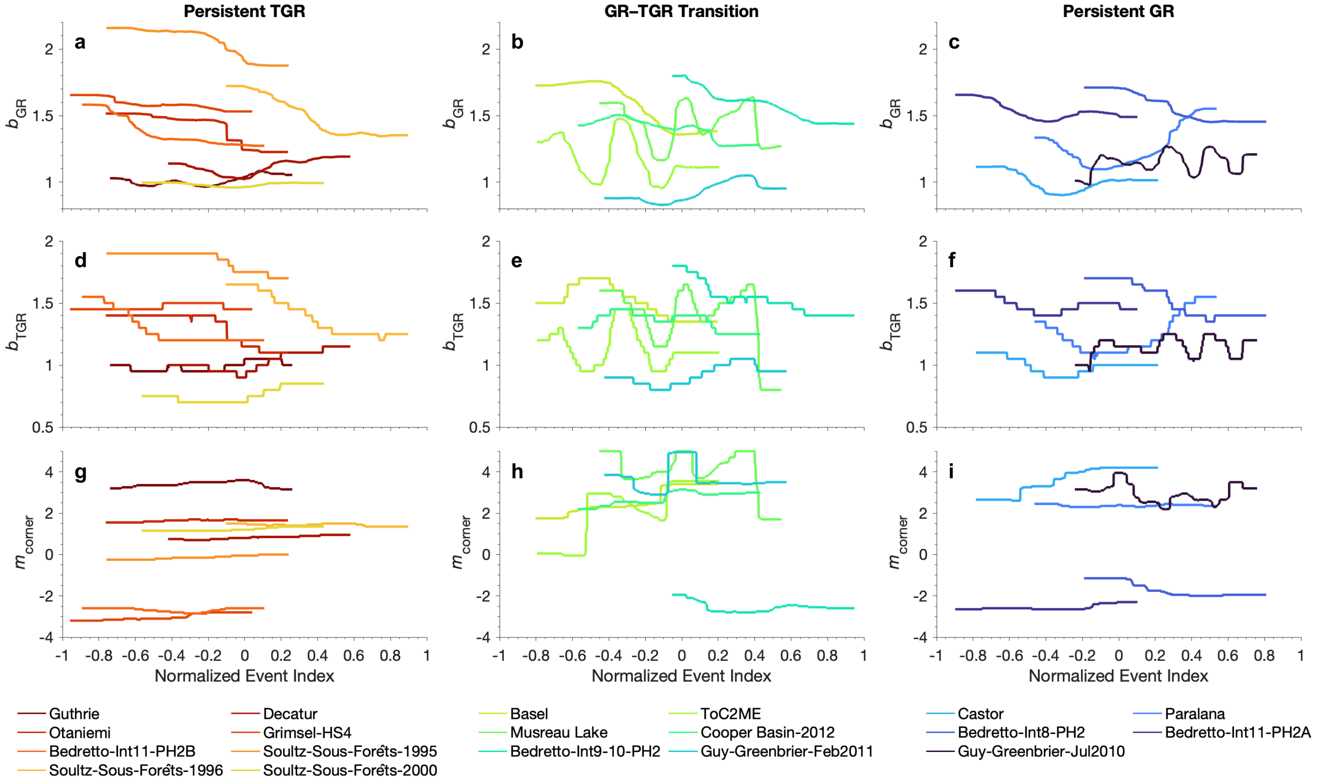

Supplementary Fig. 7 | Temporal variations in magnitude–frequency distribution parameters show no systematic pattern. The evolution of the GR *b*-value, TGR *b*-value, and corner magnitude are shown from top to bottom.

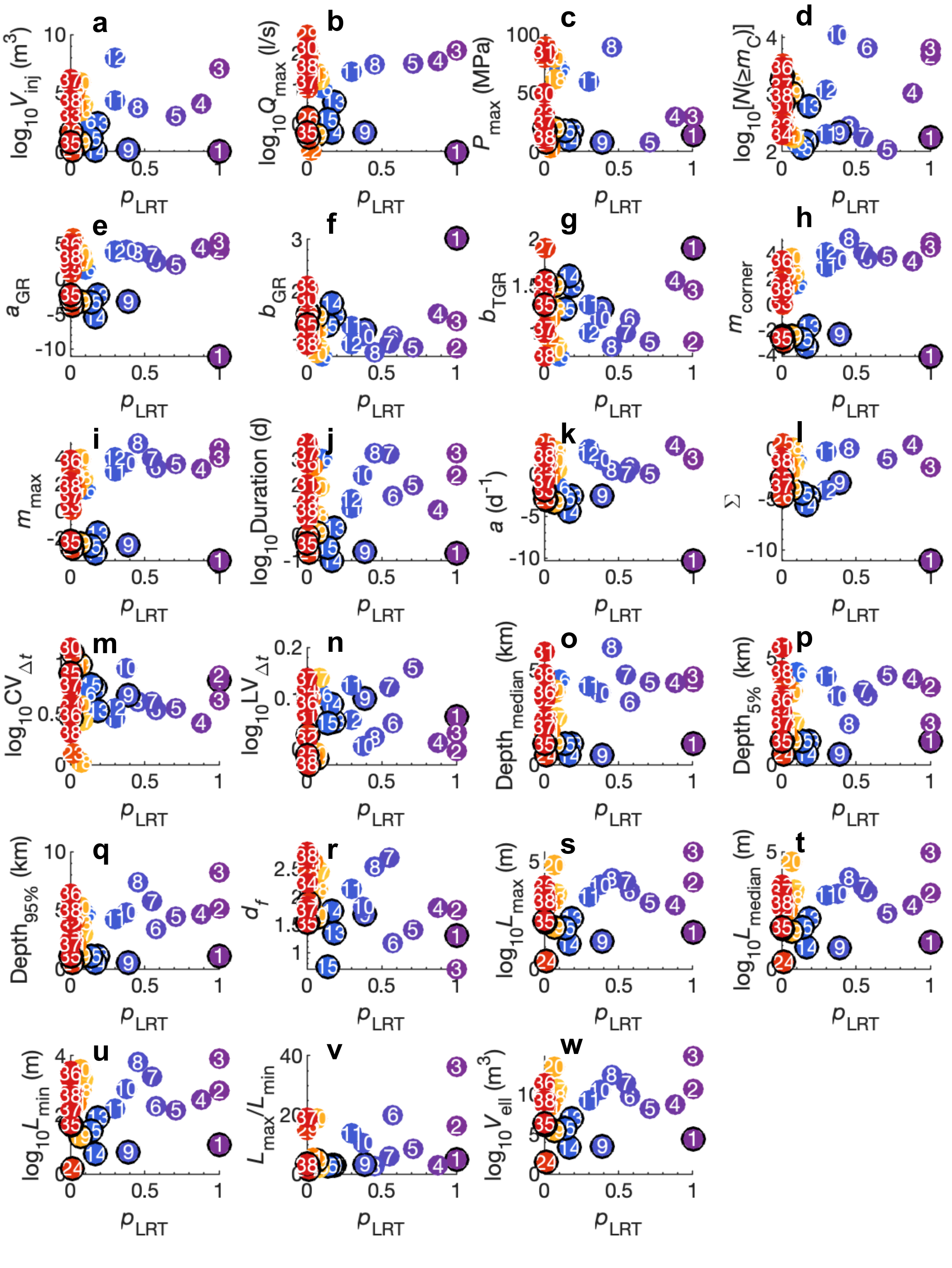

Supplementary Fig. 8 | Various quantities versus the likelihood-ratio test *p*-value. Color coding and indexing are consistent with those in Fig. 1. Circles outlined in black indicate small-scale experiments. The examined quantities include: **a,** total injected volume, **b,** peak flow rate, **c,** peak wellhead pressure, **d,** number of events above the magnitude of completeness (m_C), **e,** GR a-value, **f,** GR b-value, **g,** TGR b-value, **h,** TGR corner magnitude, **i,** observed maximum magnitude, **j,** duration of the sequence, **k,**

earthquake rate (normalized GR a-value per day), **l,** seismogenic index, **m,** global coefficient of variation of interevent time, **n,** local coefficient of variation of interevent time, **o,** the 5th, **p,** the 50th, and **q,** the 95th percentile of event depth, **r,** fractal dimension of hypocenter pair distances, **s,** the maximum, **t,** the median, and **u,** the minimum semi-axis lengths of the minimum ellipsoid that contains at least 90% of the events, **v,** the ratio between the maximum and minimum semi-axes, and **w,** the ellipsoid volume.

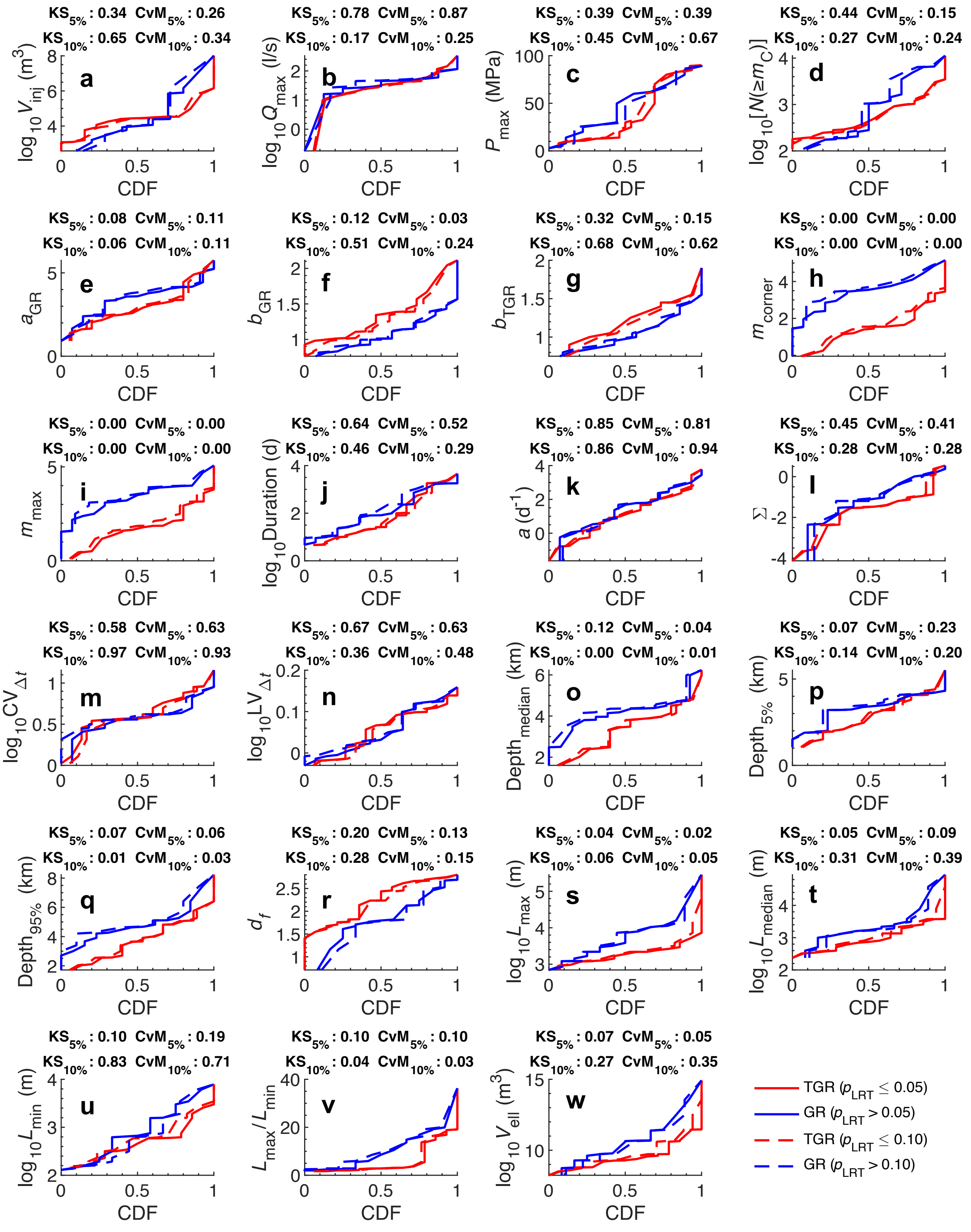

Supplementary Fig. 9 | Comparison between empirical cumulative distribution functions (CDFs) of operational and statistical quantities for tapered Gutenberg–Richter (TGR) and Gutenberg–

Richter (GR) catalogs. Empirical CDFs are shown for cases classified as TGR (red) and GR (blue), excluding small-scale experiments. The examined quantities include: **a,** total injected volume, **b,** peak flow rate, **c,** peak wellhead pressure, **d,** number of events above the magnitude of completeness (m_C), **e,** GR a-value, **f,** GR b-value, **g,** TGR b-value, **h,** TGR corner magnitude, **i,** observed maximum magnitude, **j,** duration of the sequence, **k,** earthquake rate (normalized GR a-value per day), **l,** seismogenic index, **m,** global coefficient of variation of interevent time, **n,** local coefficient of variation of interevent time, **o,** the 5th, **p,** the 50th, and **q,** the 95th percentile of event depth, **r,** fractal dimension of hypocenter pair distances, **s,** the maximum, **t,** the median, and **u,** the minimum semi-axis lengths of the minimum ellipsoid that contains at least 90% of the events, **v,** the ratio between the maximum and minimum semi-axes, and **w,** the ellipsoid volume. Both a p_{LRT} threshold of 0.05 (solid curves) and 0.10 (dashed curves) are used to distinguish between the two distributions. The p-values from the Kolmogorov–Smirnov (KS) test and the Cramér–von Mises (CvM) test are shown at the top of each panel.

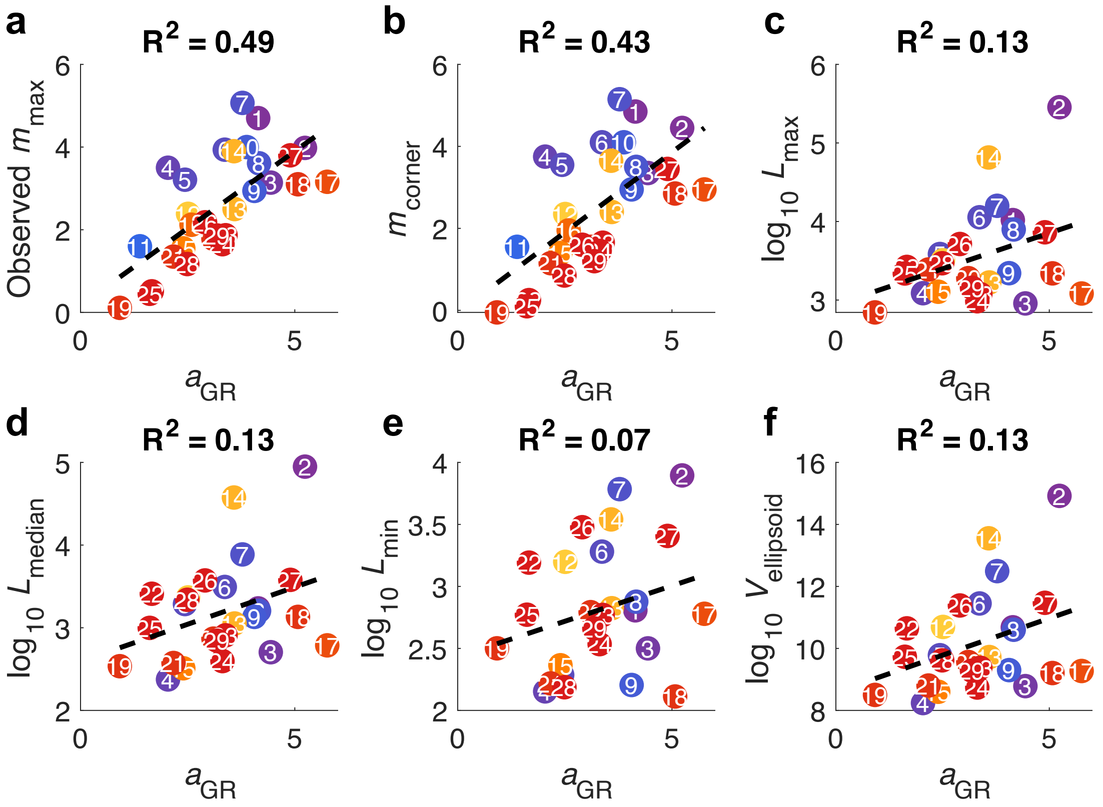

Supplementary Fig. 10 | Correlation of seismicity productivity with event size and spatial extent. Seismicity productivity is expressed as the Gutenberg–Richter a-value. The examined quantities include: **a,** observed maximum magnitude, **b,** TGR corner magnitude, **c,** the maximum, **d,** the median, and **e,** the minimum semi-axis lengths of the minimum ellipsoid that contains at least 90% of the events, **f,** the ellipsoid volume. Color coding and indexing are consistent with those in Fig. 1. Small-scale experiments are not included. Black dashed lines indicate linear fits; R^2 values show the goodness-of-fit.

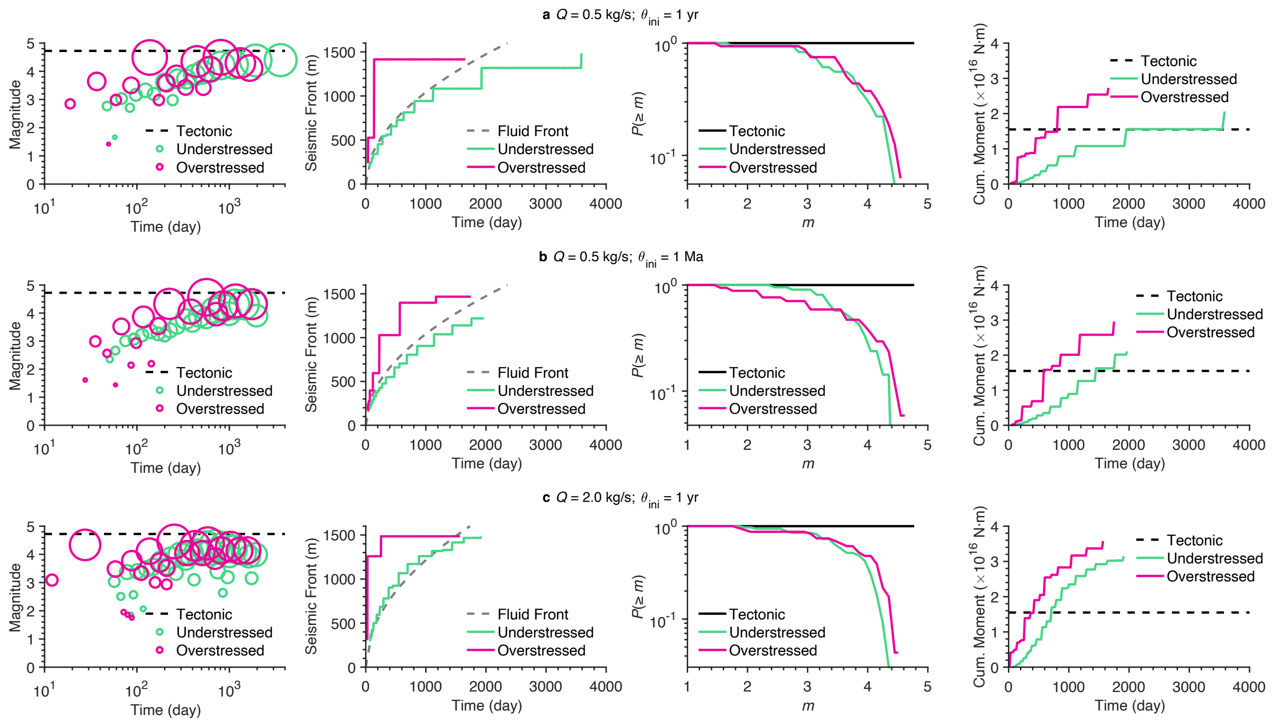

Supplementary Fig. 11 | Characteristics of simulated injection-induced earthquakes on a planar homogeneous fault. a, The benchmark simulation corresponding to Fig. 3a–d. **b**, The initial state variable, representing healing time, is set to 1 Ma instead of 1 yr. **c**,The fluid injection rate is 2 kg/s rather than 0.5 kg/s. In addition to the magnitude–time and magnitude–frequency distributions, we present the evolution of the seismic front and the cumulative seismic moment over time. The seismic front is defined as the distance from the farthest rupture point to the injection site. The dashed curve denotes the theoretical diffusion front at the 0.58 MPa contour. In the cumulative seismic moment plot, the gray dashed line represents the moment of the characteristic tectonic-driven earthquake.

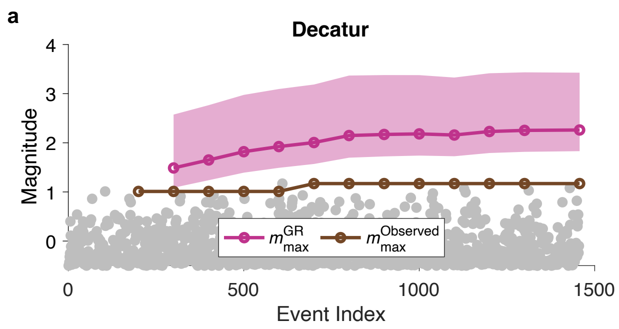

Supplementary Fig. 12 | Real-time forecast of maximum magnitude using the alternative magnitude–frequency distribution (MFD) model. Similar to Fig. 4, but maximum magnitude predictions are derived using the MFD type opposite to that selected by the likelihood-ratio test. **a,** Predicted maximum magnitude assuming a Gutenberg–Richter (GR) model for Decatur. **b,** Predicted maximum magnitude assuming a tapered Gutenberg–Richter (TGR) model for Basel.

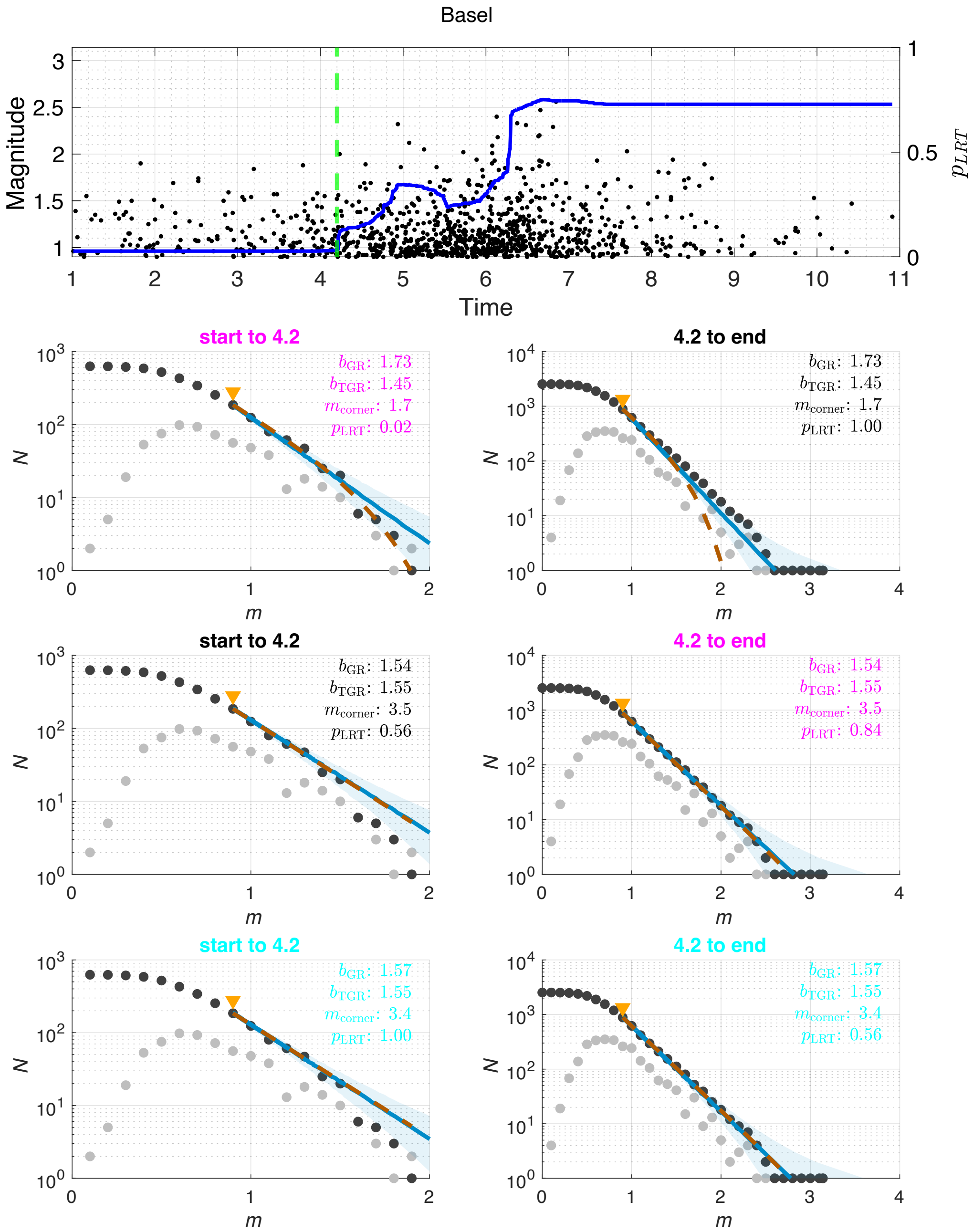

Supplementary Fig. 13 | Temporal variation of the magnitude–frequency distribution (MFD) in Basel. The top panel shows the magnitude–time series and the temporal evolution of the likelihood-ratio test p-value. Time is expressed in days. The green dashed line indicates the time at which the sequence is split into subwindows. The second, third, and last rows display the observed MFDs for earthquakes occurring before (left) and after (right) the green dashed line. The second, third, and last rows show fits of the optimal MFD models derived respectively from the first subcatalog, the second subcatalog, and the entire catalog, applied to both subcatalogs for comparison. We find that the tapered Gutenberg–Richter (TGR) model fitted to the first subcatalog does not adequately describe the second subcatalog, and conversely, the Gutenberg–Richter (GR) model fitted to the second subcatalog does not adequately describe the first subcatalog. We therefore suggest that there is a transition from a TGR to a GR distribution in this sequence.

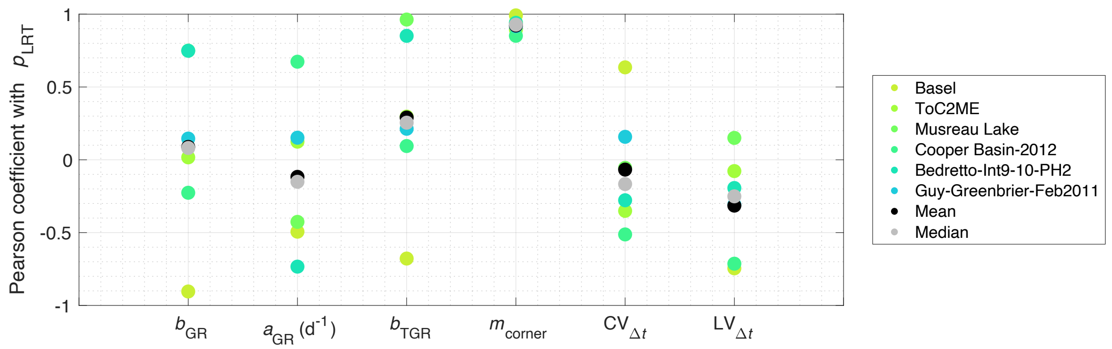

Supplementary Fig. 14 | Correlation between the likelihood-ratio test *p*-value and other indicators. The mean and median of different sequences are shown by black and gray circles, respectively.

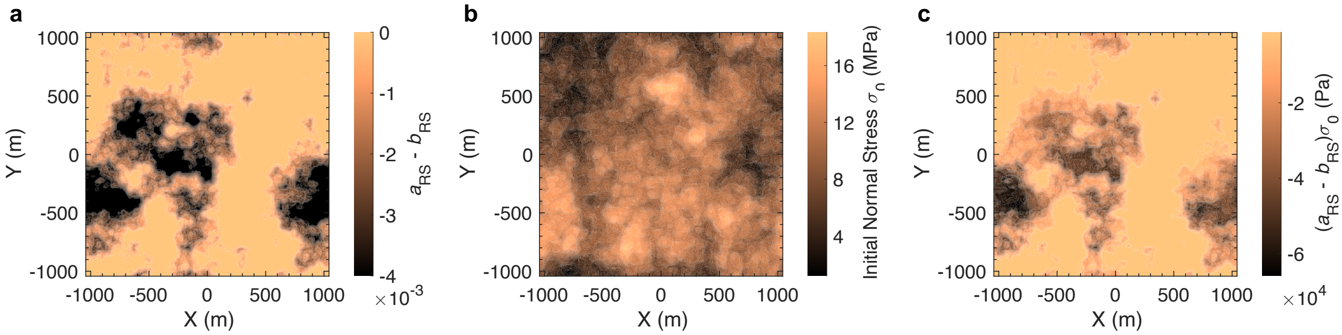

Supplementary Fig. 15 | Self-affine parameter distributions used for earthquake sequence simulations. **a**, The distributions for rate-and-state parameter $a_{RS} - b_{RS}$. **b**, The distributions for initial normal stress σ_0. **c**, The distributions for $(a_{RS} - b_{RS})\sigma_0$.

References

1 Herrmann, M., Kraft, T., Tormann, T., Scarabello, L. & Wiemer, S. A consistent high-resolution catalog of induced seismicity in Basel based on matched filter detection and tailored post-processing. *Journal of Geophysical Research: Solid Earth* **124**, 8449-8477 (2019).

2 Hager, B. H. *et al.* A process-based approach to understanding and managing triggered seismicity. *Nature* **595**, 684-+ (2021). https://doi.org:10.1038/s41586-021-03668-z

3 Luu, K., Schoenball, M., Oldenburg, C. M. & Rutqvist, J. Coupled Hydromechanical Modeling of Induced Seismicity From CO2 Injection in the Illinois Basin. *Journal of Geophysical Research: Solid Earth* **127**, e2021JB023496 (2022). https://doi.org:https://doi.org/10.1029/2021JB023496

4 Holschneider, M., Zöller, G. & Hainzl, S. Estimation of the maximum possible magnitude in the framework of a doubly truncated Gutenberg–Richter model. *Bulletin of the Seismological Society of America* **101**, 1649-1659 (2011).

5 Cornell, C. A. & Vanmarcke, E. H. in *Proceedings of the fourth world conference on earthquake engineering.* 69-83.

6 Obermann, A. *et al.* Seismic response of hectometer-scale fracture systems to hydraulic stimulation in the Bedretto Underground Laboratory, Switzerland. *Journal of Geophysical Research: Solid Earth* **129**, e2024JB029836 (2024).

7 Bröker, K. *et al.* Constraining the stress field and its variability at the BedrettoLab: Elaborated hydraulic fracture trace analysis. *International Journal of Rock Mechanics and Mining Sciences* **178**, 105739 (2024).

8 Cesca, S. *et al.* Seismicity at the Castor gas reservoir driven by pore pressure diffusion and asperities loading. *Nature Communications* **12**, 4783 (2021).

9 Li, D., Zhang, M., Zheng, J., Yang, R. & Peng, S. How induced earthquakes respond to pre-existing fractures and hydraulic fracturing operations? a case study in South China. *Journal of Geophysical Research: Solid Earth* **129**, e2024JB028691 (2024).

10 Baisch, S., Vörös, R., Weidler, R. & Wyborn, D. Investigation of fault mechanisms during geothermal reservoir stimulation experiments in the Cooper Basin, Australia. *Bulletin of the Seismological Society of America* **99**, 148-158 (2009).

11 Baisch, S. *et al.* Continued geothermal reservoir stimulation experiments in the Cooper Basin (Australia). *Bulletin of the Seismological Society of America* **105**, 198-209 (2015).

12 Bao, X. & Eaton, D. W. Fault activation by hydraulic fracturing in western Canada. *Science* **354**, 1406-1409 (2016).

13 Dando, B. *et al.* Relocating microseismicity from downhole monitoring of the Decatur CCS site using a modified double-difference algorithm. *Geophysical Journal International* **227**, 1094-1122 (2021).

14 Martínez-Garzón, P. *et al.* Spatiotemporal changes, faulting regimes, and source parameters of induced seismicity: A case study from The Geysers geothermal field. *Journal of Geophysical Research: Solid Earth* **119**, 8378-8396 (2014).

15 Villiger, L. *et al.* Influence of reservoir geology on seismic response during decameter-scale hydraulic stimulations in crystalline rock. *Solid Earth* **11**, 627-655 (2020).

16 Chen, X. *et al.* Temporal correlation between seismic moment and injection volume for an induced earthquake sequence in central Oklahoma. *Journal of Geophysical Research: Solid Earth* **123**, 3047-3064 (2018).

17 Park, Y., Mousavi, S. M., Zhu, W., Ellsworth, W. L. & Beroza, G. C. Machine-learning-based analysis of the Guy-Greenbrier, Arkansas earthquakes: A tale of two sequences. *Geophysical Research Letters* **47**, e2020GL087032 (2020).
18 Huang, Y. & Beroza, G. C. Temporal variation in the magnitude-frequency distribution during the Guy-Greenbrier earthquake sequence. *Geophysical Research Letters* **42**, 6639-6646 (2015). https://doi.org:https://doi.org/10.1002/2015GL065170
19 Friberg, P. A., Besana-Ostman, G. M. & Dricker, I. Characterization of an earthquake sequence triggered by hydraulic fracturing in Harrison County, Ohio. *Seismological Research Letters* **85**, 1295-1307 (2014).
20 Schultz, R., Park, Y., Aguilar Suarez, A. L., Ellsworth, W. L. & Beroza, G. C. En echelon faults reactivated by wastewater disposal near Musreau Lake, Alberta. *Geophysical Journal International* **235**, 417-429 (2023).
21 Cladouhos, T. T. *et al.* Results from newberry volcano EGS demonstration, 2010–2014. *Geothermics* **63**, 44-61 (2016).
22 Leonhardt, M. *et al.* Seismicity during and after stimulation of a 6.1 km deep enhanced geothermal system in Helsinki, Finland. *Solid Earth Discussions* **2020**, 1-29 (2020).
23 Albaric, J. *et al.* Monitoring of induced seismicity during the first geothermal reservoir stimulation at Paralana, Australia. *Geothermics* **52**, 120-131 (2014).
24 Woo, J. U. *et al.* An in-depth seismological analysis revealing a causal link between the 2017 MW 5.5 Pohang earthquake and EGS project. *Journal of Geophysical Research: Solid Earth* **124**, 13060-13078 (2019).
25 Kim, K.-H. *et al.* The 2017 ML 5.4 Pohang earthquake sequence, Korea, recorded by a dense seismic network. *Tectonophysics* **774**, 228306 (2020).
26 Han, J. *et al.* Research catalog of inland seismicity in the southern Korean Peninsula from 2012 to 2021 using deep learning techniques. *Seismological Research Letters* **95**, 952-968 (2024).
27 Maurer, V. *et al.* Seismicity induced during the development of the Rittershoffen geothermal field, France. *Geothermal Energy* **8**, 5 (2020).
28 Baumgartner, J., Jung, R., Gérard, A., Baria, R. & Garnish, J. The European HDR project at Soultz sous forets: Stimulation of the second deep well and first circulation experiments. (Socomine, Route de Kutzenhausen, Soultz sous Forets, FR; Bundesanstalt fur …, 1996).
29 Drif, K., Lengliné, O., Kinscher, J. & Schmitbuhl, J. Induced Seismicity Controlled by Injected Hydraulic Energy: The Case Study of the EGS Soultz-Sous-Forêts Site. *Journal of Geophysical Research: Solid Earth* **129**, e2023JB028190 (2024). https://doi.org:https://doi.org/10.1029/2023JB028190
30 Zbinden, D., Rinaldi, A. P., Diehl, T. & Wiemer, S. Potential influence of overpressurized gas on the induced seismicity in the St. Gallen deep geothermal project (Switzerland). *Solid Earth* **11**, 909-933 (2020).
31 Diehl, T., Kraft, T., Kissling, E. & Wiemer, S. The induced earthquake sequence related to the St. Gallen deep geothermal project (Switzerland): Fault reactivation and fluid interactions imaged by microseismicity. *Journal of Geophysical Research: Solid Earth* **122**, 7272-7290 (2017).
32 Schmittbuhl, J. *et al.* Induced and triggered seismicity below the city of Strasbourg, France from November 2019 to January 2021. *Comptes Rendus. Géoscience* **353**, 561-584 (2021). https://doi.org:10.5802/crgeos.71

33 Zhang, F., Wang, R., Chen, Y. & Chen, Y. Spatiotemporal variations in earthquake triggering mechanisms during multistage hydraulic fracturing in Western Canada. *Journal of Geophysical Research: Solid Earth* **127**, e2022JB024744 (2022).
34 Niemz, P., McLennan, J., Pankow, K. L., Rutledge, J. & England, K. Circulation experiments at Utah FORGE: Near-surface seismic monitoring reveals fracture growth after shut-in. *Geothermics* **119**, 102947 (2024). https://doi.org:10.1016/j.geothermics.2024.102947
35 Koirala, R. *et al.* Induced seismicity and surface deformation associated with long-term and abrupt geothermal operations in Blue Mountain, Nevada. *Earth and Planetary Science Letters* **643**, 118883 (2024).
36 Ritz, V. A. *et al.* Pseudo-Prospective Forecasting of Induced and Natural Seismicity in the Hengill Geothermal Field. *Journal of Geophysical Research: Solid Earth* **129**, e2023JB028402 (2024). https://doi.org:https://doi.org/10.1029/2023JB028402
37 Megies, T. & Wassermann, J. Microseismicity observed at a non-pressure-stimulated geothermal power plant. *Geothermics* **52**, 36-49 (2014). https://doi.org:10.1016/j.geothermics.2014.01.002
38 Li, B. Q., Khoshmanesh, M. & Avouac, J.-P. Surface Deformation and Seismicity Induced by Poroelastic Stress at the Raft River Geothermal Field, Idaho, USA. *Geophysical Research Letters* **48**, e95108 (2021). https://doi.org:10.1029/2021gl095108
39 Guo, H. *et al.* Microseismicity modulation due to changes in geothermal production at San Emidio, Nevada, USA. *Geophysical Research Letters* **52**, e2024GL112063 (2025).
40 Norbeck, J. H., McClure, M. W. & Horne, R. N. Field observations at the Fenton Hill enhanced geothermal system test site support mixed-mechanism stimulation. *Geothermics* **74**, 135-149 (2018).
41 Kwiatek, G. *et al.* Microseismicity induced during fluid-injection: A case study from the geothermal site at Groß Schönebeck, North German Basin. *Acta Geophysica* **58**, 995-1020 (2010).
42 Horner, R., Barclay, J. & MacRae, J. Earthquakes and hydrocarbon production in the Fort St. John area of northeastern British Columbia. *Can. J. Explor. Geophys* **30**, 39-50 (1994).
43 Clarke, H., Eisner, L., Styles, P. & Turner, P. Felt seismicity associated with shale gas hydraulic fracturing: The first documented example in Europe. *Geophysical Research Letters* **41**, 8308-8314 (2014). https://doi.org:10.1002/2014gl062047
44 Schultz, R. & Wang, R. Newly emerging cases of hydraulic fracturing induced seismicity in the Duvernay East Shale Basin. *Tectonophysics* **779**, 228393 (2020). https://doi.org:10.1016/j.tecto.2020.228393
45 Holland, A. A. Earthquakes Triggered by Hydraulic Fracturing in South-Central Oklahoma. *Bulletin of the Seismological Society of America* **103**, 2845-2858 (2013). https://doi.org:10.1785/0120120109
46 Goodfellow, S. D., Nasseri, M. H. B., Maxwell, S. C. & Young, R. P. Hydraulic fracture energy budget: Insights from the laboratory. *Geophysical Research Letters* **42**, 2015GL063093 (2015). https://doi.org:10.1002/2015gl063093
47 Schultz, R. *et al.* Chasing the ghost of fracking in the Vaca Muerta Formation: Induced seismicity in the Neuquén Basin, Argentina. *Seismica* **3**, 1435 (2024). https://doi.org:10.26443/seismica.v3i2.1435
48 Clarke, H., Verdon, J. P., Kettlety, T., Baird, A. F. & Kendall, J. M. Real-Time Imaging, Forecasting, and Management of Human-Induced Seismicity at Preston New Road,

Lancashire, England. *Seismological Research Letters* **90**, 1902-1915 (2019). https://doi.org:10.1785/0220190110
49 Minetto, R., Helmstetter, A., Edwards, B. & Guéguen, P. How injection history can affect hydraulic fracturing-induced seismicity: insights from downhole monitoring at Preston New Road, UK. *Bulletin of the Seismological Society of America* (2023).
50 Kwiatek, G. *et al.* Insights Into Complex Subdecimeter Fracturing Processes Occurring During a Water Injection Experiment at Depth in Äspö Hard Rock Laboratory, Sweden. *Journal of Geophysical Research: Solid Earth* **123**, 6616-6635 (2018). https://doi.org:https://doi.org/10.1029/2017JB014715
51 Jost, M. L., Büßelberg, T., Jost, Ö. & Harjes, H.-P. Source parameters of injection-induced microearthquakes at 9 km depth at the KTB Deep Drilling site, Germany. *Bulletin of the Seismological Society of America* **88**, 815-832 (1998). https://doi.org:10.1785/bssa0880030815
52 van der Elst, N. J., Page, M. T., Weiser, D. A., Goebel, T. H. & Hosseini, S. M. Induced earthquake magnitudes are as large as (statistically) expected. *Journal of Geophysical Research: Solid Earth* **121**, 4575-4590 (2016).
53 Baisch, S., Bohnhoff, M., Ceranna, L., Tu, Y. & Harjes, H.-P. Probing the Crust to 9-km Depth: Fluid-Injection Experiments and Induced Seismicity at the KTB Superdeep Drilling Hole, Germany. *Bulletin of the Seismological Society of America* **92**, 2369-2380 (2002). https://doi.org:10.1785/0120010236
54 Duboeuf, L. *et al.* Aseismic Motions Drive a Sparse Seismicity During Fluid Injections Into a Fractured Zone in a Carbonate Reservoir. *Journal of Geophysical Research: Solid Earth* **122**, 8285-8304 (2017). https://doi.org:https://doi.org/10.1002/2017JB014535
55 Priolo, E. *et al.* The Birth of an Underground Gas Storage in a Depleted Gas Reservoir—Results From Integrated Seismic and Ground Deformation Monitoring. *Earth and Space Science* **11**, e2023EA003275 (2024). https://doi.org:https://doi.org/10.1029/2023EA003275
56 Tang, L., Lu, Z., Zhang, M., Sun, L. & Wen, L. Seismicity Induced by Simultaneous Abrupt Changes of Injection Rate and Well Pressure in Hutubi Gas Field. *Journal of Geophysical Research: Solid Earth* **123**, 5929-5944 (2018). https://doi.org:https://doi.org/10.1029/2018JB015863
57 Stork, A. L., Verdon, J. P. & Kendall, J.-M. The microseismic response at the In Salah Carbon Capture and Storage (CCS) site. *International Journal of Greenhouse Gas Control* **32**, 159-171 (2015). https://doi.org:10.1016/j.ijggc.2014.11.014
58 Glubokovskikh, S. *et al.* A Small CO2 Leakage May Induce Seismicity on a Sub-Seismic Fault in a Good-Porosity Clastic Saline Aquifer. *Geophysical Research Letters* **49**, e2022GL098062 (2022). https://doi.org:https://doi.org/10.1029/2022GL098062
59 Seeber, L., Armbruster, J. G. & Kim, W.-Y. A Fluid-Injection-Triggered Earthquake Sequence in Ashtabula, Ohio: Implications for Seismogenesis in Stable Continental Regions. *Bulletin of the Seismological Society of America* **94**, 76-87 (2004). https://doi.org:10.1785/0120020091
60 Hennings, P. H. *et al.* Pore Pressure Threshold and Fault Slip Potential for Induced Earthquakes in the Dallas-Fort Worth Area of North Central Texas. *Geophysical Research Letters* **48**, e2021GL093564 (2021). https://doi.org:https://doi.org/10.1029/2021GL093564

61 Schultz, R., Stern, V. & Gu, Y. J. An investigation of seismicity clustered near the Cordel Field, west central Alberta, and its relation to a nearby disposal well. *Journal of Geophysical Research: Solid Earth* **119**, 3410-3423 (2014). https://doi.org:https://doi.org/10.1002/2013JB010836
62 Schoenball, M. & Ellsworth, W. L. Waveform-Relocated Earthquake Catalog for Oklahoma and Southern Kansas Illuminates the Regional Fault Network. *Seismological Research Letters* **88**, 1252-1258 (2017). https://doi.org:10.1785/0220170083
63 Ogwari, P. O., DeShon, H. R. & Hornbach, M. J. The Dallas-Fort Worth Airport Earthquake Sequence: Seismicity Beyond Injection Period. *Journal of Geophysical Research: Solid Earth* **123**, 553-563 (2018). https://doi.org:https://doi.org/10.1002/2017JB015003
64 Yeck, W. L. *et al.* Far-field pressurization likely caused one of the largest injection induced earthquakes by reactivating a large preexisting basement fault structure. *Geophysical Research Letters* **43**, 10,198-110,207 (2016). https://doi.org:https://doi.org/10.1002/2016GL070861
65 Yeck, W. L., Sheehan, A. F., Benz, H. M., Weingarten, M. & Nakai, J. Rapid Response, Monitoring, and Mitigation of Induced Seismicity near Greeley, Colorado. *Seismological Research Letters* **87**, 837-847 (2016). https://doi.org:10.1785/0220150275
66 Verdecchia, A., Cochran, E. S. & Harrington, R. M. Fluid-Earthquake and Earthquake-Earthquake Interactions in Southern Kansas, USA. *Journal of Geophysical Research: Solid Earth* **126**, e2020JB020384 (2021). https://doi.org:https://doi.org/10.1029/2020JB020384
67 Holland, A. A. Preliminary Analysis of the 2013 Love County Earthquake Swarm. 19 (Norman, OK).
68 Yeck, W. L., Block, L. V., Wood, C. K. & King, V. M. Maximum magnitude estimations of induced earthquakes at Paradox Valley, Colorado, from cumulative injection volume and geometry of seismicity clusters. *Geophysical Journal International* **200**, 322-336 (2014). https://doi.org:10.1093/gji/ggu394
69 Yeck, W. L. *et al.* Oklahoma experiences largest earthquake during ongoing regional wastewater injection hazard mitigation efforts. *Geophysical Research Letters* **44**, 711-717 (2017). https://doi.org:https://doi.org/10.1002/2016GL071685
70 Schultz, R. *et al.* Disposal From In Situ Bitumen Recovery Induced the ML 5.6 Peace River Earthquake. *Geophysical Research Letters* **50**, e2023GL102940 (2023). https://doi.org:https://doi.org/10.1029/2023GL102940
71 Keranen, K. M., Savage, H. M., Abers, G. A. & Cochran, E. S. Potentially induced earthquakes in Oklahoma, USA: Links between wastewater injection and the 2011 Mw 5.7 earthquake sequence. *Geology* **41**, 699-702 (2013).
72 Watkins, T. J., Verdon, J. P. & Rodríguez-Pradilla, G. The temporal evolution of induced seismicity sequences generated by low-pressure, long-term fluid injection. *Journal of Seismology* **27**, 243-259 (2023).
73 Alba, S. G., Jiménez, C. V. & Zang, A. Evidencing the relationship between injected volume of water and maximum expected magnitude during the Puerto Gaitán (Colombia) earthquake sequence from 2013 to 2015. *Geophysical Journal International* **220**, 335-344 (2020).
74 Molina, I., Velásquez, J. S., Rubinstein, J. L., Garcia-Aristizabal, A. & Dionicio, V. Seismicity induced by massive wastewater injection near Puerto Gaitán, Colombia. *Geophysical Journal International* **223**, 777-791 (2020).

75 Rubinstein, J. L., Ellsworth, W. L., McGarr, A. & Benz, H. M. The 2001–present induced earthquake sequence in the Raton Basin of northern New Mexico and southern Colorado. *Bulletin of the Seismological Society of America* **104**, 2162-2181 (2014).

76 Skoumal, R. J. *et al.* The induced Mw 5.0 March 2020 west Texas seismic sequence. *Journal of Geophysical Research: Solid Earth* **126**, e2020JB020693 (2021).

77 Hsieh, P. A. & Bredehoeft, J. D. A reservoir analysis of the Denver earthquakes: A case of induced seismicity. *Journal of Geophysical Research: Solid Earth* **86**, 903-920 (1981).

78 Wang, Z., Lei, X., Ma, S., Wang, X. & Wan, Y. Induced earthquakes before and after cessation of long-term injections in Rongchang gas field. *Geophysical Research Letters* **47**, e2020GL089569 (2020).

79 Frohlich, C. *et al.* The 17 May 2012 M4.8 earthquake near Timpson, East Texas: An event possibly triggered by fluid injection. *Journal of Geophysical Research: Solid Earth* **119**, 581-593 (2014). https://doi.org:https://doi.org/10.1002/2013JB010755

80 Kim, W. Y. Induced seismicity associated with fluid injection into a deep well in Youngstown, Ohio. *Journal of Geophysical Research: Solid Earth* **118**, 3506-3518 (2013).

81 Verdon, J. P. & Bommer, J. J. Comment on “Activation rate of seismicity for hydraulic fracture wells in the Western Canadian sedimentary basin” by Hadi Ghofrani and Gail M. Atkinson. *Bulletin of the Seismological Society of America* **111**, 3459-3474 (2021).

82 Gan, W. & Frohlich, C. Gas injection may have triggered earthquakes in the Cogdell oil field, Texas. *Proceedings of the National Academy of Sciences* **110**, 18786-18791 (2013).

83 Kwiatek, G., Bulut, F., Bohnhoff, M. & Dresen, G. High-resolution analysis of seismicity induced at Berlín geothermal field, El Salvador. *Geothermics* **52**, 98-111 (2014).

84 EPOS, I. Episode: COOPER BASIN. (2020). <https://episodesplatform.eu/#episode:COOPER_BASIN>.

85 Villaseñor, A., Herrmann, R. B., Gaite, B. & Ugalde, A. Fault reactivation by gas injection at an underground gas storage off the east coast of Spain. *Solid Earth* **11**, 63-74 (2020).
