# Supplementary material for: Large-magnitude events unlikely in induced earthquake sequences
Source: Nat Commun. 2026 Apr 29;17:4192. doi: 10.1038/s41467-026-72219-9 (PMC13153268; doi:10.1038/s41467-026-72219-9)
Supplement: Supplementary file 2 — Description of Additional Supplementary File [file 41467_2026_72219_MOESM2_ESM.pdf]

Description of Additional Supplementary Files:

Supplementary Data 1: Informa3on of all gathered injec3on-induced cases.

Supplementary Data 2 : Summary of 38 injec3on-induced cases analyzed in this study.

Supplementary Movie 1: Rupture video for tectonic sequence on a heterogeneous fault.

Supplementary Movie 2: Rupture video for induced sequence from near-field injection, starting in the early interseismic period, on a heterogeneous fault.

Supplementary Movie 3: Rupture video for induced sequence from near-field injection, starting in the late interseismic period, on a heterogeneous fault.

Supplementary Movie 4: Rupture video for induced sequence from far-field injection, starting in the early interseismic period, on a heterogeneous fault.

Supplementary Movie 5: Rupture video for induced sequence from far-field injection, starting in the late interseismic period, on a heterogeneous fault.
